# Vitamin D deficiency in healthy breastfed term infants at 3 months & their mothers in India: Seasonal variation & determinants

**Published:** 2011-03

**Authors:** Vandana Jain, Nandita Gupta, Mani Kalaivani, Anurag Jain, Aditi Sinha, Ramesh Agarwal

**Affiliations:** *Department of Pediatrics, All India Institute of Medical Sciences, New Delhi, India*; * *Department of Endocrinology & Metabolism, All India Institute of Medical Sciences, New Delhi, India*; ** *Department of Biostatistics, All India Institute of Medical Sciences, New Delhi, India*; + *Department of Radiodiagnosis, Maharaja Agrasen Hospital, New Delhi, India*

**Keywords:** Infants, lactating mothers, rickets, seasonal variation, secondary hyperparathyroidism, vitamin D deficiency

## Abstract

**Background & objectives:**

Vitamin D deficiency with a resurgence of rickets and tetany are increasingly being reported in young infants from temperate regions, African Americans and also from India. The data on vitamin D status of healthy term breastfed Indian infants and mothers are scant. Therefore, we undertook this study to determine the prevalence of vitamin D deficiency and insufficiency [serum 25 hydroxyvitamin D (25OHD) ≤ 15 ng/ml and 15-20 ng/ml, respectively] among healthy term breastfed 3 month old infants and their mothers, evaluate for clinical and radiological rickets in those infants having 25OHD < 10 ng/ml, and check for seasonal variation and predictors of infants’ vitamin D status.

**Methods:**

A total of 98 infants aged 2.5 to 3.5 months, born at term with appropriate weight and their mothers were enrolled; 47 in winter (November- January) and 51 in summer (April-June). Details of infants’ feeding, vitamin D supplementation, sunlight exposure and mothers’ calcium and vitamin D intake were recorded. Serum calcium, phosphate, alkaline phosphatase, 25 hydroxyvitamin D (25OHD) and parathormone were estimated.

**Results:**

Vitamin D deficiency was found in 66.7 per cent of infants and 81.1 per cent of mothers; and insufficiency in an additional 19.8 per cent of infants and 11.6 per cent of mothers. Radiological rickets was present in 30.3 per cent of infants with 25OHD < 10 ng/ml. 25OHD did not show seasonal variation in infants but maternal concentrations were higher in summer [11.3 (2.5 - 31) ng/ml] compared to winter [5.9 (2.5-25) ng/ml, *P*=0.003]. Intake of vitamin supplement, sunlight exposure and mother’s 25OHD were predictors of infants’ 25OHD levels.

**Interpretation & conclusions:**

Prevalence of vitamin D deficiency and insufficiency was found to be high in breastfed infants and their mothers, with radiological rickets in a third of infants with 25OHD < 10 ng/ml in this study. Studies with large sample need to be done in different parts of the country to confirm these findings.

Vitamin D is the essential precursor of 1, 25-hydroxyvitamin D, the steroid hormone required for calcium absorption, bone development and growth in children. During the first 6-8 wk of life, the vitamin D status of infants is determined by the vitamin D levels at birth, which depend on the vitamin D status of the mother^1^. Breast milk concentration of vitamin D is low (<20 IU/l) and is inadequate for the needs of the growing infant^2^. Vitamin D in breast milk relates to mothers’ vitamin D intake, skin pigmentation and sunlight exposure^3^. This implies that babies born to mothers with vitamin D deficiency are very likely to develop vitamin D deficiency unless supplemented from outside or adequately exposed to sunlight which is often not practical during early infancy.

Vitamin D deficiency with a resurgence of rickets is increasingly being reported in infants and toddlers from various parts of the world, especially from temperate regions and among African American babies^4^–^6^. Rickets and hypocalcemic seizures due to vitamin D deficiency in exclusively breastfed young infants have been recently reported from southern India^7^,^8^. There are a few reports of vitamin D deficiency among pregnant women and cord blood of their newborns^9^,^10^ and breastfed young infants from India^11^,^12^. The seasonal difference in vitamin D concentration in infants and lactating mothers, prevalence of radiological rickets among those infants with biochemical vitamin D deficiency and factors that can predict the vitamin D concentration in infants have not been reported from India.

This study was undertaken to determine the prevalence of vitamin D deficiency and insufficiency [serum 25 hydroxyvitamin D (25OHD) < 15 ng/ml and 15-20 ng/ml, respectively] among healthy breastfed term infants at the age of 3 months and their mothers and the prevalence of radiological rickets among those infants with 25OHD <10 ng/ml. Further, seasonal variation and predictors of infants’ 25OHD concentration were also determined.

## Material & Methods

The study was conducted at the All India Institute of Medical Sciences, New Delhi (28.38 N, 77.12 E with zenith angle of 84.5° in peak summer and 38.5° in peak winter) between November 2006 and March 2008. Ethics Committee of the institute approved the study protocol.

### 

#### Enrollment of study participants:

The calculated sample size for an anticipated prevalence of vitamin D deficiency of 80 per cent to fall within 10 per centof the true prevalence with 95 per centconfidence was 256. However, because of logistic constraints, it was planned to enroll 100 mother-infant dyads- 50 each born during summer and winter. Taking into consideration an up to 20 per cent loss to follow up, 120 consecutive infants born at term with weight appropriate for gestational age (>25^th^ centile for gestational age, as per Indian reference charts^13^ and their mothers were enrolled within 48 h of birth; 60 each during summer (April- June) and winter (November- January). The exclusion criteria were presence of chronic disease in mother, any gross congenital anomaly in the infant or illness requiring hospitalization in neonatal period.

After taking written informed consent from the mothers, demographic and antenatal data were recorded, counselling for exclusive breast feeding done and a date for follow up visit at the age of 10-14 wk given. Telephonic reminders were also made close to the follow up date. A total of 98 of the infants reported for follow up at the age of 10-14 wk (47 in winter and 51 in summer).

#### Clinical and anthropometric data:

Details regarding mode of feeding of infant, supplementation of any vitamin or calcium to infant, mothers’ diet, intake of calcium and vitamin D supplements during pregnancy and after delivery, education and profession, financial status of the family and usual duration and type of clothing worn during sunlight exposure for both infant and mother were recorded in a case record form. For the infants, the body surface area exposed to sunlight was calculated by assigning a score of 3 each for exposure of head and face, upper limbs, lower limbs and back and trunk making the minimum and maximum possible scores as 0 and 12, according to the method described by Ho *et al*^14^.

Mothers’ height and weight were measured and body mass index (BMI) calculated. Infant’s length, weight, and head circumference were measured and examination was done for any features of rickets.

#### Laboratory measurements:

Blood (4 ml) was drawn for estimation of serum calcium, phosphate, alkaline phosphatase (ALP), 25OHD and parathormone (PTH) for both infant and mother. Serum was separated in a refrigerated centrifuge, aliquoted, and stored at - 20°C until analyzed. Serum calcium, phosphate and ALP were measured spectrophotometrically. Serum 25OHD- levels were measured by radioimmunoassay using kits obtained from M/s DiaSorin Inc, USA and PTH levels were measured using an immunochemiluminometric assay^15^ on a Roche ELECSYS 2010 autoanalyzer.

#### Definitions:

Vitamin D deficiency was defined as 25OHD < 15 ng/ml, severe vitamin D deficiency as 25OHD <5 ng/ml and insufficiency as 25OHD 15-20 ng/ml. These definitions are based on recent recommendations regarding classification of vitamin D status in children, developed after consideration of biomarkers such as changes in ALP, bone density and calcium absorption at varying concentrations of 25OHD^16^. The cut-off for elevated PTH was taken as 46 pg/ml based on recent studies in vitamin D replete subjects^17^. Hypocalcemia was defined as serum calcium < 9 mg/dl for infants and < 8.6 mg/dl for mothers and ALP was considered elevated beyond a concentration of 420 U/l for infants and 120 U/l for mothers, respectively^18^.

#### Radiographic evaluation:

Infants with 25OHD <10 ng/ml were subjected to radiography of bilateral wrists to detect features of rickets. The radiographs were interpreted by a single radiologist, experienced in reporting of paediatric radiographs, who was blinded to the biochemical vitamin D status of the infants.

#### Statistical analysis:

Statistical analysis was carried out using STATA 9.0 (College Station, Texas, USA). Data were presented as number (%), mean ± SD/ median and inter-quartile range (IQR). The combined prevalence (95% C.I.) of vitamin D deficiency and insufficiency was presented for the primary objective. The Odds Ratio (95% C.I.) for vitamin D deficiency in infants born to mothers with vitamin D deficiency was reported. Difference in median (IQR) of infants’ 25OHD between the levels of categorical variables (season, vitamin supplement intake, type of feeding) was compared using Wilcoxon rank sum test. Spearman rank correlation was calculated to find the strength of relationship between infants’ 25OHD and sun exposure and mothers’ 25OHD. Simple followed by multiple linear regressions were used to find factors associated with infants’ 25OHD concentration after adjusting for gestational age and age at the time of sampling. Results are expressed as regression coefficient (SE) along with R^2^.

## Results

Approximately two-thirds of the infants were exclusively breastfed while the remaining also received cow or buffalo milk 1-4 times per day. A third of the infants received approximately 125 IU of vitamin D2 supplement in the form of multivitamin drops starting from 6 wk of age. The mean weight, length and head circumference of the study infants at the time of sampling corresponded to the 50^th^ centile of the WHO multicenter growth reference curves^19^. None of the infants had clinical features of rickets on examination (Table I).

**Table I:** Clinical and biochemical characteristics of infants and mothers (n=98 each)

Infants’ characteristic	Summary statistic	Mothers’ characteristic	Summary statistic

Males No. (%)	57 (58.2)	BMI^2^	23.1 ± 3.3
Birth weight (g)	2971 ± 340	Calcium supplement in pregnancy No. (%)	96 (97.9)
		Dose (mg)†	1000 (500-1000)
		Duration (wk)†	24 (16-36)
Gestational age (wk)	38.3 ± 1.0	Calcium supplement in lactation No. (%)	81 (82.6)
		Dose (mg)†	1000 (500-1000)
		Duration (wk)†	11 (2-17)
Age at sampling (wk)	13.6 ± 2.2	Working mothers No. (%)	12 (12.2)
Anthropometry at sampling		Mother’s education No. (%)	
Weight (g)	5944 ± 773	High school or less	56 (57.1)
Length (cm)	61.3 ± 2.7	Graduation or more	42 (42.9)
Head circumference (cm)	40.1± 1.2		
Exclusive breastfeeding No. (%)	69 (70.6)	Vegetarian diet No. (%)	54 (55)
Intake of vitamin supplement No. (%)	34 (34.7)	Milk intake No. (%)	
		<500 ml	67 (74.5)
		>500 ml	23 (25.5)
Sunlight exposure†		Sunlight exposure duration (min/day)†	15 (0-150)
Duration (min/day)	10 (0-120)		
Exposure score	4.8 (0-12)		
Serum calcium (mg/dl)	10.0 ± 0.8	Serum calcium (mg/dl)	9.1± 0.7
Serum phosphate (mg/dl)	6.1 ± 0.7	Serum Phosphate (mg/dl)	4.1 ± 0.8
Serum ALP (U/l)	721.1± 313.4	Serum ALP (U/l)	227.0 ± 74.0
Serum 25OHD (ng/ml)†	10.1 (2.5 - 17.1)	Serum 25OHD (ng/ml)†	9.8 (5.0 - 13.8)
Serum PTH (pg/ml)†	42.5 (29.9 - 83.6)	Serum PTH (pg/ml)	49.2 (37.9 - 74.9)

ALP, alkaline phosphatase; 25OHD, 25 hydroxyvitamin D; PTH, parathormone

† Data presented as median (IQR), otherwise mean ± SD

All mothers had received regular antenatal care at our hospital, belonged mostly to Hindu middle income family and resided in urban/suburban area. Nearly all reported taking a combined supplement containing 500 mg of calcium and 250 IU of vitamin D3, once or twice per day in pregnancy. This was continued postnatally for varying periods of time by a majority of the mothers. All mothers habitually wore clothing that allowed exposure of head, face and arms and did not practice ’purdah’ or use sunscreen.

**Fig. 1:**
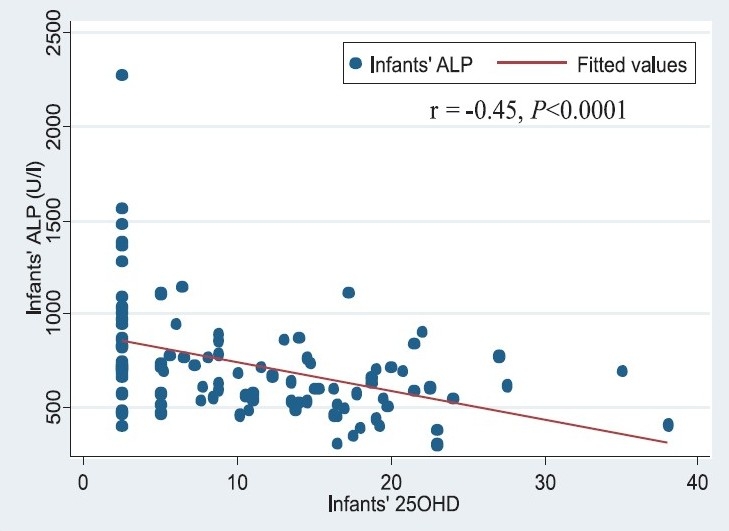
Scatter plot describing the relationship between infants’ serum 25OHD and alkaline phosphatase (ALP) concentrations (r= -0.45, *P*< 0.001).

**Fig. 2:**
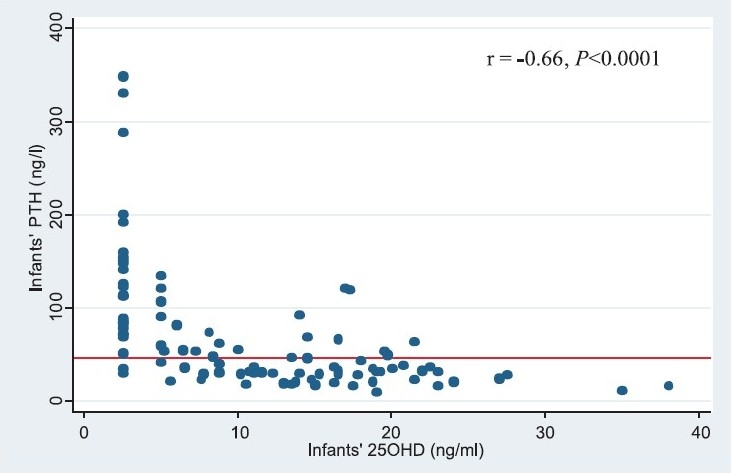
Scatter plot describing the relationship between infants’ serum 25OHD and PTH concentrations (r= -0.66, *P*< 0.001).

Mean calcium and phosphate concentrations were normal in infants as well as mothers. However, the mean concentration of ALP was markedly raised above the upper limits of normal in infants as well as the mothers. The prevalence of the biochemical markers of vitamin D deficiency in infants and mothers is presented in Table II. The combined prevalence (95% C.I.) of vitamin D deficiency and insufficiency (25OHD < 20 ng/ml) in infants and mothers was 86.5 per cent (78.0, 92.6) and 92.6 per cent (85.4, 97.0), respectively. There was a significant inverse correlation between infants’ 25OHD and ALP (r=-0.45, *P*<0.0001) (Fig. 1). Among infants and mothers with vitamin D deficiency, hyperparathyroidism was present in 62.5 and 57.0 per cent, respectively. Amongst those with severe vitamin D deficiency, hyperparathyroidism was present in 90.3 per cent infants and 73.1 per cent mothers. There was a strong inverse correlation between serum 25OHD and PTH concentration in the infants, (r = -0.66, *P*<0.001) (Fig. 2) as well as in mothers (r = -0.25, *P*<0.01).

**Table II:** Prevalence of vitamin D deficiency and its biochemical markers in infants and mothers (n=98)

Parameter	Infants n (%)	Mothers n (%)

Vitamin D deficiency	64 (66.7)	77 (81.1)
Vitamin D insufficiency	19 (19.8)	11 (11.6)
Severe vitamin D deficiency	26 (27.1)	22 (23.2)
Hypocalcemia	7 (7.1)	23 (24.2)
Raised ALP	89 (91.8)	92 (96.8)
Hyperparathyroidism	47 (48.5)	52 (53.7)

ALP, alkaline phosphatase; 25OHD, 25 hydroxyvitamin D; PTH, parathormone

Radiographs of bilateral wrists could be obtained for 33 of 48 infants with serum 25OHD < 10 ng/ ml. Radiological evidence of rickets was seen in 10 (30.3%) infants. This was in the form of metaphyseal widening in 7 infants and healing line in 3 infants. The infants with radiological rickets had lower levels of serum 25OHD, calcium and phosphate and higher ALP compared to those without, although not statistically significant.

Serum 25OHD concentration did not show significant seasonal variation in infants (summer 10.5 (2.5-27.5) ng/ml, winter 8.8 (2.5-38) ng/ml. However, in mothers, concentration was significantly higher in summer at 11.3 (2.5-31) ng/ml compared to that in winter of 5.9 (2.5-25) ng/ml, *P*=0.003. In the infants, duration of exposure to sunlight in winter was significantly higher at 60 (30-120) minutes compared to that in summer of 0 (0-5) min; *P*< 0.05.

**Fig. 3:**
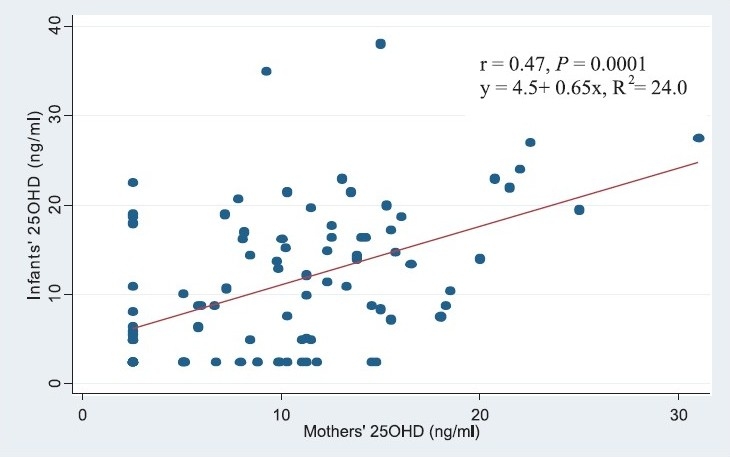
Regression of infants’ and maternal serum 25OHD concentration.

On bivariate analysis, infants who were on vitamin supplement had significantly higher serum 25OHD [13.6 (2.5-38) ng/ml] compared to those who were not [8.6 (2.5-27.5) ng/ml, *P*= 0.009]. The result of linear regression analysis is summarized in Table III Mothers’ 25OHD concentration, infants’ sunlight exposure (product of duration and exposure score) and infants’ vitamin supplement intake were found to be statistically significant independent predictors of infants’ 25OHD concentration. The relation between infants’ and mothers’ 25OHD concentration is presented in Fig. 3.

**Table III:** Linear regression analysis for predictors of infants’ 25OHD (ng/ml)

Variable	Univariate			Multivariate*		
	β (SE)	*P* value	R^2^	β (SE)	*P* value

Vitamin supplement	4.80 (1.65)	0.005	0.082	3.52 (1.45)	0.025
Sunlight exposure	0.006 (0.002)	0.009	0.075	0.008 (0.002)	0.001
Mothers’ 25OHD	0.65 (0.12)	0.0001	0.240	0.61 (0.117)	0.0001

* R^2^= 0.43 after adjusting for gestational age and age at sampling

The odds ratio (95% CI) for vitamin D deficiency among infants born to mothers with deficiency was 3.4 (1.2, 9.9) compared to those born to mothers without deficiency.

## Discussion

We found a high prevalence of vitamin D deficiency in term, appropriate for gestational age breastfed infants and their mothers. This is much higher than the prevalence reported in studies from the United States^20^,^21^ but consistent with the findings of studies from other countries like Greece, UAE and Pakistan^22^–^24^ and with previous Indian studies^9^–^12^.

The reason for this high prevalence in India may be related to decreased cutaneous synthesis owing to higher skin pigmentation; and lower duration as well as surface area exposed to the sun due to greater coverage of body and lesser participation in outdoor activities, in particular among girls, starting from adolescence. In a recent study of schoolgirls from India, 90.8 per cent were found to have vitamin D deficiency^25^. The traditional custom of giving infants an oil massage in the sunlight for 15-30 min before bathing has gradually declined, especially in the cities. The intake of vitamin D is inadequate as food items (except infant formula) are not fortified and there is no policy of routine vitamin D supplementation in pregnant/lactating women and infants. Indian diet, low in calcium and high in phytates, may also contribute by causing secondary hyperparathyroidism, increased conversion of 25OHD to polar metabolites and inactive 24, 25 dihydroxyvitamin D3^26^,^27^.

In the present study, the physiological relevance of hypovitaminosis D is corroborated by an almost ubiquitously elevated ALP concentration; and hyperparathyroidism in half the study population. In a third of infants with 25OHD < 10 ng/ml, radiological rickets was already present, even though clinical rickets or growth retardation was absent. This was similar to the earlier findings of metaphyseal fraying in 7.5 per cent and demineralization in 32.5 per cent of healthy infants and toddlers with 25OHD < 20 ng/ml, compared to clinical rickets in only 2.5 per cent^21^.

We found no seasonal variation in the vitamin D levels of the infants even though there was a significant difference in the mothers’ levels. This is in contrast to previous studies that have found higher levels during summer^20^,^22^. This finding can be explained by the fact that the infants’ sun exposure was lower because of temperatures reaching up to 45°C during summer months. Alternative explanation can be that these infants accrued lower stores of vitamin D *in utero* as the latter half of pregnancy was in winter season.

Intake of vitamin supplement by the infant, sunlight exposure and maternal 25OHD levels were found to have positive correlation with the infants’ 25OHD. This suggests that either or all of these three strategies should be effective in raising the infants’ 25OHD. The current recommendation of American Academy of Pediatrics is that all infants and children, including adolescents, should have a minimum daily intake of 400 IU of vitamin D beginning soon after birth^28^. This may however, be inadequate. Zeghoud *et al*^29^ have shown that supplementation with 1000 but not 500 IU/day of ergocalciferol for a month could normalize the PTH levels in infants with subclinical vitamin D deficiency.

Sensible sun exposure can provide an adequate amount of vitamin D3. It is estimated that whole body exposure to 1 minimal erythemal dose is equivalent to ingesting between 10,000 and 25,000 IU of vitamin D2^30^. In an older study from United States, it was estimated that in breastfed young infants, sunlight exposure of 30 min/wk in a diaper can raise the serum 25OHD to th ≥ 11 ng/ml^3^. However, for any given infant, it is difficult to determine reliably the amount of sunlight exposure sufficient to maintain normal vitamin D status, as it is influenced by many factors such as clothing, skin pigmentation, sunscreen use, weather conditions, latitude, air pollution, time of the day and time of the year^31^. Additionally, the possible association of malignant melanoma in adulthood with degree of sunlight exposure in childhood^32^ is of concern, so much so, that the Committee on Environmental Health of the American Academy of Pediatrics has recommended that infants <6 months of age should be kept out of direct sunlight^33^.

In the United States, the currently recommended adequate intake for vitamin D during pregnancy as well as lactation is 200 IU/day^34^. However, this recommendation is not supported by data and recent studies suggest that the ideal dose should be much higher^35^,^36^.

In conclusion, the present study found a very high prevalence of vitamin D deficiency in healthy term born infants at the age of 3 months and their mothers in winter as well as summer. Secondary hyperparathyroidism was present in half of the study population, rickets in a third of the infants with 25OHD < 10 ng/ml and elevated ALP in more than 90 per cent of the infants and mothers. Maternal vitamin D status, intake of vitamin supplement by infant and exposure to sunlight were positively correlated with infants’ 25OHD concentration. Larger studies and meta-analysis should be conducted to confirm the findings of the present study and develop recommendations for vitamin D supplementation for pregnant and lactating women and young infants.
